# P-drug concept and the undergraduate teaching

**DOI:** 10.4103/0253-7613.45160

**Published:** 2008

**Authors:** Navyug Raj Singh

**Affiliations:** Pharmacology Department, Govt. Medical College, Amritsar, Punjab, India. E-mail: navyug69@gmail.com

This is in response to the letter by Khilnani G titled “The concept of personal drugs in the undergraduate pharmacology practical curriculum.”[1] For sometime now, there is an ongoing discussion on P-drug concept. Various authors have suggested that undergraduates should be sensitized toward the rational drug use and P-drug concept.[2,3]

We have been teaching P-drug concept in our institution based on the “Guide to Good Prescribing”.[4] Teaching methodology involves power-point presentations, group discussions, and problem-solving approaches. Based on this experience, some of the issues that came up during our sessions are discussed here.

“Guide to Good Prescribing” has recommended four criteria for comparison of drugs as, efficacy, safety, cost, and suitability. Students, most of the times, tend to restrict efficacy to the text book definition “maximum response produced by a drug”, although in this context, efficacy is a wider term including pharmacokinetic and pharmacodynamic characteristics of the drug. Students get confused by this term and hence it could be replaced by a wider term like, say, effectiveness.

As regards the cost, it is the total cost of treatment that is important rather than the unit price. This fact has been highlighted by Khilnani G,[1] while comparing metronidazole and tinidazole. For lifelong treatment, per day cost may be a useful tool. Students find this criterion easiest to handle and compare. Similarly, comparison of safety profile does not pose much challenge to the students.

Suitability is a criterion that requires some time before the students imbibe it satisfactorily. We tell the students to keep it at the tail end and focus on it only after they are done with other three criteria, although suitability encompasses the rest three criteria as well. Suitability refers to whether or not a drug is suitable for a given population or a given patient as regards its efficacy, safety, as well as cost. Suitability takes into account the convenience of dosage form, dosage schedule, and route of administration. It also considers the safety features like contraindications and drug interactions. Lastly, it considers the socioeconomic status of individual patient before prescribing.

It is wrong to say that suitability should not be considered during compilation of P-drug list.[2] Some features of the drugs like convenience of dosage form, dosage schedule, route of administration, etc. can always be compared while compilation.[4]

For ranking the drugs based on four criteria, different methods can be adopted. We have used various methods and found the process of comparing easy by allocating numbers to the drugs on a scale of 0–10 for each criterion. Adding up all the numbers for a drug gives the final rank conveniently.

As mentioned by Khilnani G, it is difficult to ignore the advice of clinical teachers and experts while compiling the P-drug list,[1] but it is also important that at the end of the day the students should be able to critically analyze all the available information and use it to select their own P-drugs.

Argument of Khilnani G over ‘P-drug’ verses ‘Drug of choice’ requires some deliberation.[1] P-drugs chosen by a practitioner are essentially the ‘drugs of choice’ for common conditions, according to his/her own judgment and interpretation. Why should the procedure for choosing P-drugs be different from that of drug of choice? Of course, many physicians will be inclined to choose albendazole as their P-drug for roundworm infestation, although some of them may rather choose mebendazole. We have witnessed such conflicts among our students many times, when two drugs closely compete with each other as drugs of choice.

Different authors seem to argue over the choice of a particular drug for a given condition.[1,2] If we see this in right perspective, the whole purpose of the exercise is defeated by such argument. The basic motive while teaching the concept is that the exercise should focus on “How to prescribe” rather than “What to prescribe”. This has been repeatedly stressed upon by the WHO publication also.
